# Feasibility of urinary microRNA profiling detection in intrahepatic cholestasis of pregnancy and its potential as a non-invasive biomarker

**DOI:** 10.1038/srep31535

**Published:** 2016-08-18

**Authors:** Li Ma, Xiao-Qing Zhang, Da-Xue Zhou, Yue Cui, Lin-Lin Deng, Ting Yang, Yong Shao, Min Ding

**Affiliations:** 1 Key Laboratory of Clinical Laboratory Diagnostics (Ministry of Education of China), College of Laboratory Medicine, Chongqing Medical University, Chongqing, 400016, P. R. China; 2 Biomedical Analysis Center, Third Military Medical University, Chongqing, 400030, P. R.China; 3 Department of Obstetrics and Gynecology, The First Affiliated Hospital of Chongqing Medical University, Chongqing, 400016, P. R. China

## Abstract

Intrahepatic cholestasis of pregnancy (ICP), a pregnancy-related liver disease, leads to complications for both mother and fetus. Circulating microRNAs (miRNAs) have emerged as candidate biomarkers for many diseases. So far, the circulating miRNAs profiling of ICP has not been investigated. To assess the urinary miRNAs as non-invasive biomarkers for ICP, a differential miRNA profiling was initially analyzed by individual quantitative reverse transcriptase polymerase chain reaction (qRT-PCR) assay in urinary samples from a screening set including 10 ICP and 10 healthy pregnancies. The selected candidate miRNAs were then validated by a validation set with 40 ICP and 50 healthy pregnancies using individual qRT-PCR assay. Compared with the expression in urine of healthy pregnant women, the expression levels of hsa-miR-151-3p and hsa-miR-300 were significantly down-regulated, whereas hsa-miR-671-3p and hsa-miR-369-5p were significantly up-regulated in urine from ICP patients (p < 0.05 and false discovery rate < 0.05). A binary logistic regression model was constructed using the four miRNAs. The area under the receiver operating characteristic curve was 0.913 (95% confidence interval = 0.847 to 0.980; sensitivity = 82.9%, specificity = 87.0%). Therefore, urinary microRNA profiling detection in ICP is feasible and maternal urinary miRNAs have the potential to be non-invasive biomarkers for the diagnosis of ICP.

Intrahepatic cholestasis of pregnancy (ICP), a pregnancy-related syndrome in which the incidence varies geographically from 0.1% to 15.6%^1^,^2^, is characterized by mild to severe pruritus and disturbed liver function tests. The disease symptoms and liver dysfunction appear mainly in the late second or third trimester of pregnancy and recover within a few days (typically 1–3) after delivery. However, ICP leads to complications for both mother and fetus. ICP is associated with intractable pruritus and high predisposition to postpartum bleeding, being the leading causes of maternal morbidity. On the other hand, ICP is associated with an increased risk of spontaneous preterm labor, fetal distress and sudden intrauterine death. Currently, the exact cause of ICP is unknown. Genetic, endocrinologic, nutritional, and environmental factors are considered to be related to the pathogenesis of the disease^1^,^3^,^4^,^5^. The diagnosis of ICP should exclude other clinical entities which are included in the differential diagnosis of cholestasis and hepatic disease. Viral hepatitis, autoimmune liver disease, gall bladder stones, tumors of the hepatobiliary tract, and other causes with elevated hepatic enzymes specified to pregnancy (e.g., namely preeclampsia and acute fatty liver) should be considered in the differential diagnosis^4^,^5^,^6^. ICP is related to abnormalities in the metabolism and disposition of bile acid compositions. The elevated serum total bile acids (TBA) level is considered as the most useful laboratory indicator for the diagnosis of ICP^7^. However, it was impossible to distinguish the ICP patients with low pruritus from normal pregnant women by serum TBA^8^. Further more, normal serum TBA concentrations have been observed in some cases with ICP^9^.

MicroRNAs (miRNAs) are a class of small noncoding RNAs, approximately 19–25 nucleotides, which negatively regulate gene expression and have a critical role in many biological and pathological processes^10^. miRNAs, as important post-transcription regulators, inhibit protein translation or stimulate transcript degradation though sequence-specific base pairing on the 3’untranslated regions of the target mRNAs. So miRNAs influence crucial cell processes such as differentiation, proliferation/growth, apoptosis suggesting their relevant role in human disease^11^,^12^. Besides tissue-specific expression of miRNAs, circulating mRNAs have been detected in most extracellular fluids, particularly in plasma/serum^13^,^14^. Moreover, these circulating miRNAs are highly stable and easily detected and usually reflect a tissue specific injury or expression^15^.

So far, circulating miRNAs are used as biomarkers for various forms of acute and chronic liver disorders, such as drug-induced liver injury^16^, chronic viral hepatitis^17^,^18^, hepatocellular carcinoma^19^ and nonalcohol-related fatty liver disease^20^. The search for miRNAs that might serve as biomarkers for pregnancy complications was initially started by Chim and colleagues in 2008^21^. Luo *et al*.^22^ demonstrated that miRNAs are exported to the circulation via exosome and suggested that circulating trophoblast derived miRNAs reflected the physiological status of the pregnancy and could be used diagnostically. Subsequently, placental-specific miRNAs were identified in plasma and serum in several other studies^23^,^24^. Urine, as a readily available compartment and a non-invasive source for circulating miRNAs, has been used to diagnose in many diseases^17^,^25^. So far, the circulating miRNAs profile of ICP, as a special type of cholestasis in pregnancy, has not been investigated. In the present study, we systematically screened urine miRNAs expression profiling of normal pregnant women and pregnant women with ICP by individual quantitative reverse transcriptase polymerase chain reaction (qRT-PCR) assay. We also performed validations by using individual qRT-PCR assay, in order to identify the potential biomarker of miRNAs for diagnosis of ICP.

## Results

### Patient description

The characteristics of participants are summarized in Table 1. The cases and controls were well matched for age and gestational week. However, the serum TBA, alanine transaminase (ALT), aspartate transferase (AST), alkaline phosphatase (ALP) and gamma-glutamyl transpeptidase (GGT) of patients with ICP were significantly different from those of the normal controls.

### Differential urinary miRNA profiling in ICP and healthy pregnancies

To estimate whether there was a difference in the generalizable urinary miRNA signatures between ICP and healthy pregnancies, we employed individual qRT-PCR assays to test 348 mature human miRNAs of 10 ICP and 10 healthy pregnancies (Supplementary dataset). Comparing the ICP and the control group, 24 miRNAs presented significant differential expression levels. Among them, 15 miRNAs were up-regulated (p < 0.05) and 9 were down-regulated (p < 0.05) in the ICP group. The candidate miRNAs selected for validation included 24 differential expression miRNAs and other 8 miRNAs that did not have statistically significant differential expression but |log2FC| were more than 1.5 (Table 2).

### Validation of miRNA differential expression profiling for diagnosis of ICP

**Figure 1:**
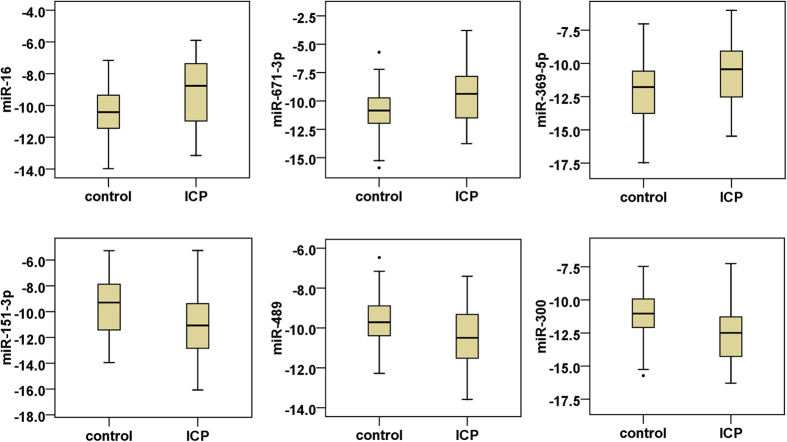
The differential expression levels of urine miRNAs in patients with ICP versus healthy pregnancies. The expression of candidate miRNAs was confirmed by individual qRT-PCR in an independent cohort of 90 (50 control vs 40 ICP) urine samples. The expression levels of hsa-miR-369-5p, hsa-miR-16, hsa-miR-671-3p, hsa-miR-151-3p, has-miR-489 and hsa-miR-300 were significantly different between ICP and control groups (p < 0.05and FDR < 0.05). The values are normalized by geometric mean normalization of *cel-miR-1832* and *cel-miR-5594-5p*, shown as relative expression at y-axis. The upper and lower limits of the boxes and the lines inside the boxes indicate the 75^th^ and 25^th^ percentiles and the median, respectively. Upper and lower horizontal bars denote the 90^th^ and 10^th^ percentiles.

**Figure 2:**
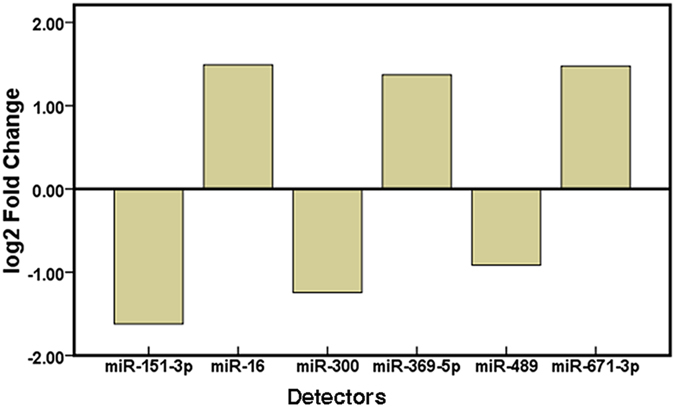
The fold change (FC) of miRNA expression in ICP urine sample relative to the expression in normal. The log2 (FC) between ICP cases and healthy pregnancies is displayed in the Y-axis. Columns: ICP urine samples. Baseline: urine samples from healthy pregnancies.

The expression of 32 candidate miRNAs selected from the previous step was confirmed by individual qRT-PCR in an independent cohort of 90 (50 control vs. 40 ICP) urine samples. The fold change of miRNA expression in ICP urine sample relative to the average expression in normal was calculated by the equation 2^−∆∆Ct^ and parametric moderated t-test was used to compare miRNA expression between patients with ICP and controls. Nine of the 32 miRNAs presented significantly different expression levels between the ICP and control group and six miRNAs of nine passed the FDR correction (FDR < 0.05) using adjusted FDR bonferroni-hochberg method. The six differential expression miRNAs between the ICP and control group are shown in Fig. 1 and the fold change (FC) of these miRNA expression is shown in Fig. 2. Compared with the expression in urine of healthy pregnant women, the expression levels of hsa-miR-151-3p, has-miR-489, and hsa-miR-300 were significantly down-regulated, whereas hsa-miR-16, hsa-miR-671-3p, and hsa-miR-369-5p were significantly up-regulated in urine of ICP patients.

**Figure 3:**
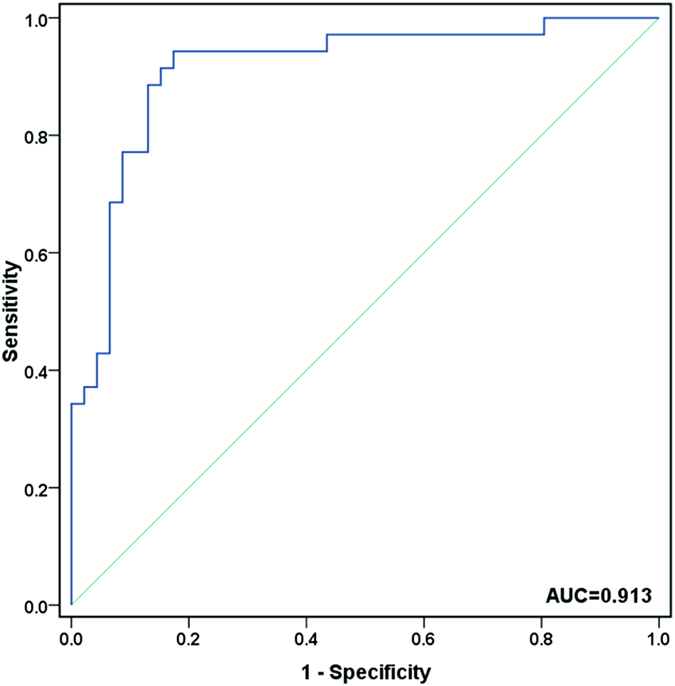
ROC curve analysis for discrimination between the cases of ICP pregnancies and healthy pregnancies.

A binary logistic regression model to estimate the risk of being diagnosed with ICP was applied to the validation set (50 controls vs. 40 ICP urine samples). Four of the six miRNAs turned out to be significant predictors (threshold: enter variable, if p < 0.05; remove variable, if p > 0.1). The predicted probability of being diagnosed with ICP was determined by the four miRNAs (hsa-miR-151-3p, hsa-miR-671-3p, hsa-miR-369-5p and hsa-miR-300) panel logit model and used to construct the receiver operating characteristic (ROC) curve. The miRNA panel area under the curve (AUC) was 0.913 (95%CI = 0.847 to 0.980; sensitivity = 82.9%, specificity = 87.0%, Fig. 3).

## Discussion

Circulating miRNAs were reported to serve as an improved biomarker for several diseases^19^,^26^,^27^,^28^ because of their robustness against severe conditions that would normally degrade most RNAs, such as enzymatic degradation, freezing, and thawing, or extreme pH conditions^14^,^29^. In addition to robustness, easy access is another important criterion for biomarkers. miRNAs are detectable in almost all body fluids and excretions, including urine, feces, saliva, tear, ascitic, pleural, and amniotic fluid^13^,^15^,^30^, which could provide a new set of diagnostic tools for a variety of diseases.

ICP is associated with an increased risk of spontaneous preterm labor, fetal distress and sudden intrauterine death. Therefore, an accurate and early diagnosis of ICP is essential^31^. Usually, diagnosis of ICP is based on pruritus with abnormal liver function^32^. However, pruritus in pregnancy is a common symptom but it could be the only evidence in ICP. Moreover, it is often difficult to make an accurate diagnosis by performing solely routine laboratory tests because liver function is also altered in some other conditions of pregnant women. Serum TBA more than 10 μmol/L is considered as one of the most important standards in diagnosing ICP^7^. Five pregnant women in our study who were finally diagnosed as ICP after delivery had serum TBA less than 10 μmol/L. The five patients were misdiagnosed using serum TBA as the biomarker. Fortunately, only one patient was misdiagnosed using the miRNA panel. The circulating miRNAs profile has not been investigated so far. However, the circulating miRNAs profiling of pregnancy-specific disease and in primary biliary cirrhosis (PBC), a cholestatic disease had been reported. A prospective longitudinal cohort study was designed by Hromadnikova *et al*. to predict subsequent onset of gestational hypertension. The result showed that the up-regulation of miR-516-5p, miR-517^*^, miR-520 h and miR-518b was associated with a risk of later development of gestational hypertension^33^. Ura B *et al*. found serum miR-1233 might represent a potential marker of early sPE^34^. However, a research by Luque *et al*. showed maternal serum miRNA assessment at first-trimester of pregnancy does not appear to have any predictive value for early preeclampsia^23^. A multistage retrospective nested case-control study designed by ChunZhao *et al*. showed serum miRNAs (miR-29a, miR-222 and miR-132) are differentially expressed between GDM women and controls and could be candidate biomarkers for predicting GDM. Due to the significant limitations of currently available noninvasive tools to diagnose and monitor liver damage in various forms of liver diseases, and growing evidence for a prominent role of specific miRNA in liver pathobiology, research about circulating miRNA profiling in liver disease are rapidly growing^35^. For cholestatic disease, available data on circulating miRNAs and PBC appear in the literature in recent study. Recently, a human study in PBC patients showed that the expression of miR-505-3p and 197-3p in serum was reduced in PBC patients compared with healthy controls and patients with viral hepatitis^36^. Tan *et al*. found that miR-122-5p, miR-144-3p, and miR-26b-5p in serum were with high diagnostic accuracy for PBC^37^.

As the same as tissue-specific miRNA profiling determination, qRT-PCR and its variations are the most frequently used techniques for circulating miRNA profiling test in body fluids. However, enrichment before the analysis is a crucial step for the circulating miRNAs expression profiling. A common problem that may limit the quantity of miRNAs accessible for expression analysis is their stability and resistance to storage handling. Fortunately, circulating miRNAs are resistant to severe conditions. Accordingly, our study explored urinary miRNA profiling in ICP patients by individual qRT-PCR both for screening and validation stage. Compared with the expression in urine of healthy pregnant women, the expression levels of hsa-miR-151-3p and hsa-miR-300 were significantly down-regulated, whereas hsa-miR-671-3p and hsa-miR-369-5p were significantly up-regulated in urine of ICP patients. MiR-151, as an oncogenic miRNA hosted by the FAK gene, is associated with various forms of cancer with a tendency to be up-regulated^38^. Even though miR-151–3p expression was found to be decreased in the development of cardiac hypertrophy^39^, peripheral blood mononuclear cells of schizophrenia patients^40^. and polycystic ovary syndrome patient follicular fluid^41^. miR-671-3p has been certified to be associated with MMP-9 gene polymorphism in different carcinoma^42^. miR-300 was found to be down-regulated in cancer cells that have undergone epithelial–mesenchymal transition comparing with typical epithelial phenotype carcinoma cells^43^. Meanwhile, miRNA-369-5p was up-regulated in mesenchymal cells compared with epithelial cells in endometrial carcinosarcomas series^44^. Yabushita *et al*.^45^ identified miRNA-369-5p was significantly increased in the serum of pancreatic ductal adenocarcinoma rats compared to that in the control rats. An inverse correlation between expression of miR-369-5p and its target gene was observed by Z. Xu *et al*.^46^ miR-369-5p was down-regulated in DSA^+^ LT while its gene targets in TGF-β signal pathways were up-regulated. TGF-β may be involved in the pathogenesis of ICP through inhibiting the production of TNF-α and IFN-γ^46^.

In conclusion, with this pilot trial we demonstrate the feasibility to detect an ICP dependent miRNA profiling in urine. The test enables us to specifically discriminate patients with ICP from healthy pregnant women. We could identify four significantly altered and specifically regulated miRNAs (hsa-miR-151-3p, hsa-miR-671-3p, hsa-miR-369-5p and hsa-miR-300) in ICP patients from those in healthy controls. Our present results show typical expression patterns of miRNA in the urine of ICP patients. This sustains the potential role of urinary miRNAs as non-invasive innovative biomarkers in diagnosis of ICP. Since this study examines only a limited number of samples, extended future studies are needed to confirm these observations.

## Methods

### Research subjects and design

We designed case-control study, including urine from 50 cases of ICP and 60 cases of healthy pregnant women to find the maternal circulating miRNAs for diagnosis of ICP. This study was performed at the First Affiliated Hospital of Chongqing Medical University, Chongqing, and designed conformed to the ethics guidelines given in the Declaration of Helsinki. Written informed consent was obtained from all subjects and all experimental protocols were approved by the ethics committee of the First Affiliated Hospital of Chongqing Medical University. Maternal urine samples were obtained from pregnancies who received prenatal care at the third-trimester (≥28 gestational weeks) from October 2013 to August 2015. The enrollment criteria for ICP were as follows: pruritus and jaundice in the third trimester of pregnancy without signs of chronic liver diseases, skin diseases, or symptomatic cholelithiasis; elevated levels of aminotransferases and total serum bile acid; and normalization of cholestasis after delivery. For the control group, only those healthy pregnant women matched with cases on age and pregnant weeks were enrolled during the same period, excluding women with a history of gallstones or cholecystopathy, pruritus, drugs consumption, hepatitis, or any other diseases damaging hepatobiliary function. Women with ICP underwent fetal monitoring from the time of diagnosis until delivery, and received drug therapy such as ursodeoxycholic acid (UDCA) for symptom relief. Samples from the women with ICP were collected at the first visit to confirm diagnosis before drug treatment. Serum liver function tests, including serum liver function tests, including TBA, TBIL, DBIL, ALT, AST, ALP, and GGT were performed on an automated Olympus Chemistry Analyzer (AU5400, Olympus, Japan).

### Sample collection and miRNA extraction

The urine samples were obtained and processed within 2 h. Processing involved centrifugation at 3,000 g for 10 min at 4 °C, followed by centrifugation at 12,000 g for 10 min at 4 °C. The supernatant was removed and stored at −80 °C until analysis. The miRNA from urine was isolated using the QuantoBio’s microRNA Purification Kit (QuantoBio Biotek Corporation, Beijing, China) according to the manufacturer’s protocol. In brief, 300 μL of lysis buffer was added to 200 μL of urine sample. The sample was mixed in a tube followed by adding 550 μL of acidified phenol:chloroform. After mixing vigorously for 30 s, the sample was then centrifuged at 15,000 g for 10 min at 4 °C. The 200 μL supernatant was carefully transferred to a new collection tube, and 500 μL volume of ethanol containing binding buffer from the kit was added and mixed. The sample was then applied directly to a silica membrane containing column and the miRNA was retained and cleaned by using wash buffer provided in the kit. The immobilized cleaned miRNA was then eluted from the membrane into a collection tube with RNase-free water. The quantity and purity of the RNA was evaluated by A260/A280 ratio using a Nanodrop 2000c spectrometer (Thermo Fisher Scientific, USA). The prepared miRNA samples were stored at −80 °C.

### Reverse transcription

The miRNA of isolation was reversely transcribed into cDNA using the Quanto-miR cDNA Synthesis Kit (QuantoBio Biotek Corporation, Beijing, China) according to the manufacturer’s protocol. In brief, *E. coli* polyA polymerase was used to add adenines at the 3′ end of RNA molecules lacking a polyA tail. After oligodT annealing, a universal tag was attached to the 3′ end of cDNAs during cDNA synthesis. With the addition of this universal tag, individual miRNAs were detected with miRNAs-pecific forward primers and a reverse universal primer mix. These cDNA samples were stored at −80 °C.

### Quantitative realtime-PCR

A SYBR Green-based real-time PCR method was used to quantify the relative expression of miRNAs. In the miRNA expression profiling array, a total of 348 human mature miRNAs were evaluated in urine of ICP and healthy pregnant women. qRT-PCR was carried out on ViiA7 (Life technology, USA) in a total reaction volume of 10 μL (including 8 μL SYBR Green I mix, 1 μL primer, and 1 μL reverse transcription product) according to the manufacturer’s protocol (QuantoBio Biotek Corporation, Beijing, China). The Echo 550 liquid handler (Labcyte, USA) was used for liquid handling. A robot (HighRes, USA) integrated PCR and liquid handler was used in the qPCR analysis. The reactions were initiated in a 384-well optical plate at 95 °C for 5 min, followed by 40 cycles of 95 °C for 15 s and 60 °C for 45 s.

### Data analysis

The expression of miRNA profile and candidate miRNA were normalized by global expression level normalization and two geometric mean normalization of two external standards (synthetic *C.elegans* miRNA *cel-miR-1832* and *cel-miR-5594-5p*) added in the steps of RNA isolation and reverse transcription, respectively. Differential miRNA expression was determined by parametric moderated t-test and corrected by bonferroni-hochberg false discovery rate (FDR), both with significance level set at 0.05. The relative expression levels of target miRNAs were calculated based on the threshold cycle (CT) value. The fold change (FC) of miRNA expression in ICP urine sample relative to the average expression in normal was the equation 2^−ΔΔCT^, in which ΔΔCT = (CTmiRNA − CTnormalization) ICP − (CTmiRNA -CTnormalization) control.

### Statistical Analysis

The characteristics of participants were compared between controls and ICP women using independent samples-t tests. Binary logistic regression was utilized to screen out the combination biomarker for diagnosis of ICP. All the statistical analyses were performed with SPSS Statistics. v17.0.0 (SPSS, Inc., Chicago, USA). P value less than 0.05 was considered statistically significant, and all tests were two tailed.

## Additional Information

**How to cite this article**: Ma, L. *et al*. Feasibility of urinary microRNA profiling detection in intrahepatic cholestasis of pregnancy and its potential as a non-invasive biomarker. *Sci. Rep.* **6**, 31535; doi: 10.1038/srep31535 (2016).

## Supplementary Material

Supplementary Information

## Figures and Tables

**Table 1:** Main clinical characteristics of the study groups.

Variables	Screening set		*P* value	Validation set		*P* value
	Controls (n = 10)	ICP (n = 10)		Controls (n = 50)	ICP (n = 40)	
Maternal age, y	28.80 ± 2.53	29.30 ± 5.56	0.060	28.93 ± 3.40	28.89 ± 4.10	0.940
Gestational week	31.80 ± 1.75	32.00 ± 3.02	0.080	32.36 ± 2.51	34.00 ± 2.15	0.110
TBA, μmol/L	2.76 ± 0.73	22.32 ± 14.66	0.004	2.67 ± 1.36	26.77 ± 21.11	0.000
TBIL, μmol/L	7.73 ± 3.17	12.69 ± 6.50	0.117	7.60 ± 2.64	10.46 ± 5.63	0.004
DBIL, μmol/L	1.79 ± 1.28	3.43 ± 2.11	0.122	1.74 ± 0.95	5.28 ± 5.03	0.000
ALT, IU/L	13.20 ± 4.37	54.73 ± 67.82	0.006	15.98 ± 17.72	88.70 ± 99.79	0.000
AST, IU/L	16.80 ± 2.66	41.40 ± 45.29	0.003	18.76 ± 9.69	53.18 ± 49.27	0.000
ALP, IU/L	96.00 ± 26.42	154.70 ± 112.46	0.004	98.18 ± 29.20	168.98 ± 100.01	0.000
GGT, IU/L	13.20 ± 10.68	31.60 ± 35.04	0.137	14.61 ± 7.93	30.11 ± 26.34	0.000

y: years; the statistical method is independent samples-t test.

**Table 2:** The miRNAs selected for validation.

miRNA	log_2_FC	*P* value	miRNA	log_2_FC	*P* value
miR-369-5p	2.73	9.03E-04	miR-30a	−1.05	3.59E-02
miR-93*	−2.46	8.13E-03	miR-369-3p	1.45	3.70E-02
miR-219-3p	2.21	8.27E-03	miR-671-3p	1.36	3.77E-02
miR-328	−1.51	9.42E-03	miR-527	1.39	4.02E-02
miR-371-3p	1.45	1.41E-02	miR-526a	1.30	4.18E-02
miR-29b	−1.52	1.49E-02	miR-26a-2*	−1.99	4.69E-02
let-7e	2.23	1.86E-02	miR-584	1.19	4.79E-02
miR-524-5p	2.14	1.88E-02	miR-183*	0.85	4.90E-02
miR-151-3p	−1.60	2.03E-02	miR-340-3p	−2.13	8.28E-02
miR-16	1.53	2.60E-02	miR-520 g	−2.11	1.52E-01
miR-658	1.66	2.66E-02	miR-98	−1.78	1.21E-01
miR-489	−1.90	2.79E-02	miR-300	−1.63	6.71E-02
miR-106b	1.53	2.82E-02	miR-302b	−1.57	1.54E-01
miR-99b	−1.55	2.82E-02	miR-204	−1.53	1.68E-01
miR-450a-5p	−3.02	3.29E-02	miR-199b-5p	2.14	5.63E-02
miR-623	1.37	3.39E-02	miR-409-5p	1.58	6.17E-02
